# Maternal 25-hydroxyvitamin D and its association with childhood atopic outcomes and lung function

**DOI:** 10.1111/cea.12172

**Published:** 2013-09-16

**Authors:** A K Wills, S O Shaheen, R Granell, A J Henderson, W D Fraser, D A Lawlor

**Affiliations:** 1 MRC Centre for Causal Analyses in Translational Epidemiology, University of BristolBristol, UK; 2 School of Social and Community Medicine, University of BristolBristol, UK; 3 Centre for Primary Care and Public Health, Blizard Institute, Barts and The London School of Medicine and Dentistry, Queen Mary University of LondonLondon, UK; 4 Norwich Medical School, University of East AngliaUK

**Keywords:** asthma, atopy, mother-offspring, vitamin D, 25(OH)D

## Abstract

**Background:**

It has been suggested that maternal vitamin D status in pregnancy influences the risk of asthma and atopy in the offspring. The epidemiological evidence to support these claims is conflicting and may reflect chance findings and differences in how vitamin D was assessed.

**Objective:**

To examine the association between blood total maternal 25-hydroxy vitamin D (25(OH)D) concentrations in pregnancy and offspring asthma, atopy and lung function in the largest birth cohort study to date.

**Methods:**

Participants were largely of white European origin and resident in the South West of England. We examined the associations of maternal 25(OH)D concentrations in pregnancy with the following outcomes in the offspring: wheeze, asthma, atopy, eczema, hayfever, at mean age 7.5 years (*n* = 3652–4696 depending on outcome), IgE at 7 years (*n* = 2915) and lung function and bronchial responsiveness at mean age 8.7 years (*n* = 3728–3784).

**Results:**

Sixty-eight per cent of mothers had sufficient (> 50 nmol/L) concentrations of 25(OH)D, 27% were insufficient (27.5–49.99 nmol/L) and 5% were deficient (< 27.5 nmol/L). There was no evidence to suggest that maternal 25(OH)D concentration in pregnancy was associated with any respiratory or atopic outcome in the offspring. These findings remained after adjustment for season of measurement and for potential confounders. There was also no evidence that these relationships followed a non-linear form and no evidence that either deficient or high concentrations of maternal 25(OH)D were associated with atopic or respiratory outcomes.

**Conclusions:**

We found no evidence that maternal blood 25(OH)D concentration in pregnancy is associated with childhood atopic or respiratory outcomes.

## Introduction

Vitamin D may play an important role in normal fetal lung and immune system development 1 and there is increasing speculation that maternal vitamin D status in pregnancy might be causally linked to the development of asthma and atopy in the offspring. It has been argued that maternal vitamin D deficiency might, at least in part, explain the rising prevalence of asthma and allergic disease in some countries 2. However, an opposing argument holds that the increasing prevalence of asthma and atopy may be partly a consequence of the fortification of childhood foods with vitamin D and hence high vitamin D status in early childhood 3–7.

Studies of maternal dietary intake have mostly reported inverse relationships between maternal vitamin D intake in pregnancy and early childhood wheeze, asthma, allergic rhinitis and eczema 8–10. However, a major limitation of these studies is that diet makes a relatively minor contribution to total vitamin D status, which is derived largely from photosynthesis in the skin. Hence, the value of analysing dietary vitamin D intake alone as a means of effectively establishing the influence of vitamin D on outcomes has been questioned as it leads to considerable misclassification of total exposure. The gold standard measure of vitamin D status is the blood concentration of 25-hydroxyvitamin D (25(OH)D) 11. Findings from studies using maternal 5, 12, 13 or cord blood 14, 15 concentrations of 25(OH)D have been conflicting. Three recent studies from the United Kingdom, United States and Spain that examined its association with childhood asthma were null 12, 13, 15, though one reported a U-shaped association between cord blood 25(OH)D and IgE and aeroallergens 15. One study from New Zealand reported a negative association with asthma 14, but another UK study reported a positive association – a higher risk of childhood asthma and wheeze among offspring born to mothers with the highest 25(OH)D concentrations (> 75 nmol/L) 5. Factors such as residual confounding, chance results attributable to small sample sizes and differences in how vitamin D exposure and childhood outcomes were measured (i.e. whether by dietary intake estimation or blood concentrations), may explain some of this between study heterogeneity.

Given these inconsistent findings, we have investigated whether maternal 25(OH)D concentrations during pregnancy are associated with atopic and respiratory outcomes in the offspring in a large population-based birth cohort study. Ours will be the largest and most comprehensive (in terms of the range of related outcomes assessed) study to date of these associations.

## Materials and methods

### Participants

**Fig. 1:**
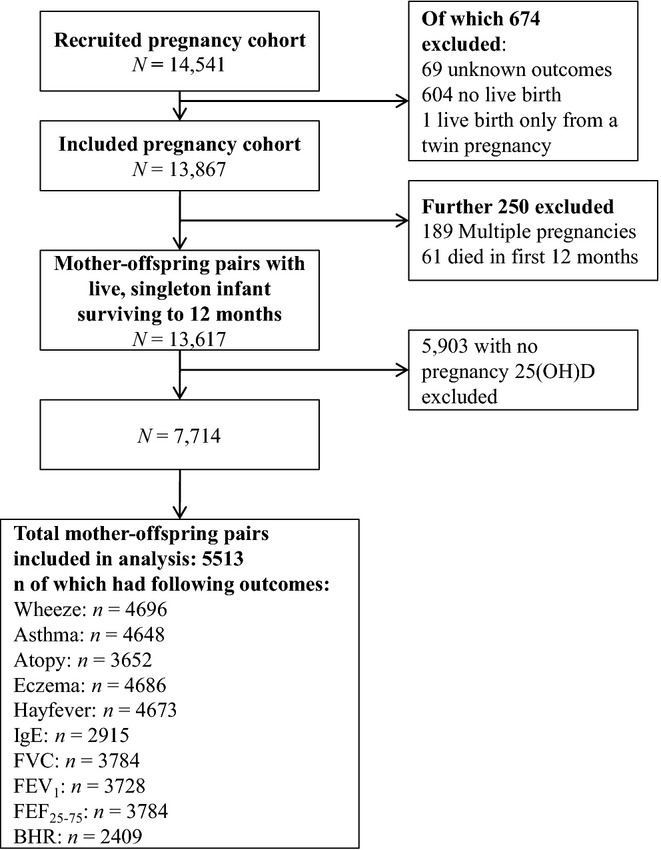
Flow chart of participants used in these analyses.

The Avon Longitudinal Study of Parents and Children (ALSPAC) is a prospective population-based study that recruited a cohort of 14 541 pregnancies resident in the South West of England with expected dates of delivery from 1st April 1991 to 31st December 1992 (http://www.alspac.bris.ac.uk.) 16, 17. A total 13 678 singleton live born infants resulted from these pregnancies. Figure 1 shows the flow of participant through the study to the eligible sample for this study. Only singleton live born offspring whose mother had a valid pregnancy 25(OH)D concentration and who had data on at least one outcome were considered eligible (*N* = 5515). Ethical approval for this study was obtained from the ALSPAC Law and Ethics Committee and the Local Research Ethics Committee.

### Outcomes

When the children were 7.5 years old, the mothers were asked: ‘Has your child had any of the following in the past 12 months: wheezing; asthma; eczema; hayfever?’ Children were defined as having current doctor-diagnosed asthma (primary outcome of interest) if mothers responded positively to the question: ‘Has a doctor ever actually said that your study child has asthma?’ and positively to one or both of the questions on wheezing and asthma in the past 12 months. Forty-four per cent of children fulfilling this definition of asthma demonstrated bronchial hyperresponsiveness as defined by a fall in forced expiratory volume in 1 s (FEV1) of 20% or more following methacholine challenge up to and including the maximum dose PD20 (1.2 mg) and 52% were atopic.

Atopy at 7 years was defined as a positive reaction (defined as a weal diameter ≥ 2 mm) to *Dermatophagoides pteronyssinus*, cat or grass (after subtracting saline reactions from histamine and allergen weals and excluding children unreactive to 1% histamine). Serum total IgE was measured by fluoroimmunoassay using the Pharmacia UNICAP system (Pharmacia & Upjohn Diagnostics AB, Uppsala, Sweden).

Pulmonary function was measured at the 8-year clinic at mean age 8.7 years (SD: 0.27) using a Vitalograph 2120 electronic spirometer with a computer-based on-screen incentive (Vitalograph, Maids Moreton, UK). The tests adhered to American Thoracic Society criteria for standardization and reproducibility of flow-volume measurement, with the exception of ATS recommendations for duration of expiration 18 as many children did not fulfil the forced expiratory time > 6 s end of test criteria, a minimal volume change over the final second was used. Outcomes included FEV_1_, FVC, FEF_25–75_ adjusted for gender, age and height and expressed as standard deviation score (SDS). Following baseline spirometry, children whose FEV_1_ was > 70% predicted for height had a bronchial challenge test with methacholine using the method of Yan et al. 19. Doubling doses of methcholine were administered at 1 min intervals to a maximum cumulative dose of 1.2 mg and expressed for each subject as the dose–response slope of FEV_1_ (percentage decline from baseline). Bronchial hyperresponsiveness (BHR) was defined as being in the highest tertile of the dose–response slope.

### Assessment of maternal and offspring 25(OH) D concentrations

Maternal non-fasting blood samples taken as part of routine antenatal care were collected and stored initially at −20°C and then at −80°C, with no further freeze–thaw cycles to the time of 25(OH)D assessment. Serum used to assess 25(OH)D could be from any stage in pregnancy and hence we have participants with samples from the first, second or third trimester, and a small number with repeat measurements of 25 (OH)D concentration (4.8%). For those with a repeat sample, we took the last available measure because the majority of mothers had samples taken in the final trimester (52%). The date of sampling was recorded and this was used, together with the date of birth and gestational age to determine gestational length at the time of 25(OH)D assessment and also to adjust for seasonality of measurement. Offspring serum 25(OH)D concentration was assessed from samples obtained at mean age 9.8 years (standard deviation: SD 1.11). As detailed previously 20, 25(OH)D concentrations in both mothers and offspring were measured in the same laboratory using high-performance liquid chromatography–tandem mass spectrometry with internal standard in a laboratory meeting the performance target set by the Vitamin D External Quality Assessment Scheme Advisory Panel. For these analyses, we use total 25(OH)D, further details of this method are provided in the Online Repository.

### Potential confounders and mediators

We considered the following as potential confounders because of their known or potential plausible association with both maternal concentrations of vitamin D and with childhood atopic and lung function outcomes 21, 22: maternal factors during pregnancy [smoking, infections, anxiety score, antibiotic use, paracetamol use, body mass index (BMI) and alcohol intake], other maternal factors (educational level, housing tenure, financial difficulties, ethnicity, age, parity, history of asthma, eczema, rhinoconjunctivitis, migraine) and postnatal factors that may reflect a background socio-economic confounding pathway originating prior to birth (breastfeeding, day care, pets, exposure to damp/mould in home, environmental tobacco smoke exposure and number of younger siblings). The following were considered as potential mediators of the relationship of outcomes with maternal 25(OH)D: gestational age, birth weight, head circumference, birth length, BMI at age 7 years and offspring serum 25(OH)D. Gender and offspring age was also adjusted for in all analysis because of their association with the outcomes in this study. More details on how these covariables were measured and classified are in Table S1 of the Online Repository.

### Statistical analysis

#### Derivation of 25(OH)D exposures

The main source of 25(OH)D_3_ [and hence total 25(OH)D] is synthesis in the skin in response to UVB exposure, which means there is a strong sinusoidal pattern of 25(OH)D with date of blood collection (see Figure S1 in the Online Repository). As bloods were sampled at random gestational lengths during pregnancy, to make the measures more comparable across mothers, we adjusted each woman's measure of 25(OH)D to the date corresponding to their respective 3rd trimester midpoint (34 weeks) using a sine–cosine regression model. We chose the 3rd trimester because the majority of mothers were sampled in this trimester. This variable incorporates the seasonal influence on 25(OH)D. To remove this seasonal influence in an attempt to obtain a marker of habitual vitamin D concentrations for each mother, we used the residuals from the sine–cosine regression model. Similar trigonometric models were fitted in the offspring to adjust for season as just described. More details on the derivation of these variables are provided in the Online Repository.

#### Main analyses

Logistic regression was used for binary outcomes and linear regression for continuous outcomes. IgE was log transformed to reduce positive skewness. Three main sets of regression models were considered: first a minimally adjusted model, adjusting for maternal age and offspring sex; second a confounder-adjusted model, adjusting for socio-economic variables, housing conditions, lifestyle behaviours, illnesses during pregnancy, ethnicity, and several other maternal characteristics; and third a model adjusting for these confounders plus additional adjustment for the seasonal pattern of maternal 25(OH)D. This last model was fitted in two steps, first the seasonal trend in maternal 25(OH)D was removed (as explained above), and this resulting variable was then used as the exposure in regression models. Where we found a suggestive association (suggestive in terms of a dose–response trend across quintiles of exposure) with 25(OH)D and an outcome, we fitted additional models with further adjustment for potential mediators, first, adjusting for gestational age and size at birth (birth weight, birth length, head circumference) and size at 7 years (BMI), and second, additionally adjusting for the offspring's seasonally adjusted 25(OH)D levels. Online Repository Table S1 details the variables in each of these models. Maternal 25(OH)D was modelled using quintiles to allow us to explore non-linearity, as suggested by others 15. Wald tests were used to perform tests of general association vs. the null hypothesis of no association.

#### Additional analyses

To test whether associations were driven by a threshold effect of greater risk in those whose mothers had insufficient levels of vitamin D, we examined associations of categories of 25(OH)D levels (≥ 50 nmol/L; 27.5–49.99 nmol/L and < 27.5 nmol/L) with outcomes, we also repeated analyses using 75 nmol/L as a higher threshold for sufficiency as recommended in a more recent review 23. As a sensitivity analysis we also estimated the association between maternal 25(OH) D and longitudinal wheeze phenotypes from birth to 7 years. These phenotypes were extracted using latent class models and distinguish between types of wheeze based on persistence of symptoms 24 (see Online Repository). Lastly, as airway development occurs in the first trimester, we tested the interactions between maternal 25(OH)D concentration and the trimester of blood sample (categorized as a binary variable indicating 1st trimester) in models of pulmonary function. Here, each mother's 25(OH)D concentration was adjusted to the midpoint of their respective trimester of measurement (using the models described above). If the effect of maternal 25(OH)D was stronger in the first trimester, then this would provide some evidence of a causal effect around a sensitive developmental period because we would expect maternal 25(OH)D to be similarly confounded across trimesters.

#### Dealing with missing data

To increase efficiency and minimize selection bias, we used multivariate multiple imputation 25 to impute missing values of covariables for eligible participants (see also Online Repository). We also repeated analyses including only those with complete data on all each outcome and covariables. Generally, the results were qualitatively similar, with slightly larger standard errors and less power (see Figures S11 and S12 in the Online Repository where sample sizes are also provided).

## Results

### Sample description and dataset

Of the 5513 maternal–offspring pairs included in this study (Fig. 1) 1397 (25%), 1223 (22%) and 2893 (52%) of the mothers had 25(OH)D assessed in the first, second and third trimester, respectively, and 2884 (52%) of the offspring were male. The majority of the mothers had sufficient levels of 25(OH)D (≥ 50 nmol/L: 68%) and only a small proportion were deficient (< 27.5 nmol/L: 5%) (Table 1 and Figure S3). Similarly, among offspring at mean age 9 years, few had deficient (1.3%) or insufficient levels of 25(OH)D (21.0%). The prevalence of reported wheeze and asthma in this cohort at 7 years was approximately 10% (Table 1).

**Table 1:** Distributions of main outcomes and exposures among those included in analysis (observed data) and others available from the eligible cohort (singletons surviving beyond 1 year, but who did not have both a maternal 25(OH)D measure and an outcome measure)

	Included in analysis sample (observed)		Others from eligible cohort		P*
		
	*N*	*n* (%) or median (IQR)	*N*	*n* (%) or median (IQR)	
Serum 25(OH)D (nmol/L)†					
Mother‡					
< 27.5		257 (4.7%)		41 (6.4)	
27.5 to 49.9		1489 (27.0%)		815 (32.3)	
≥ 50	5513	3767 (68.3%)	2203	3018 (61.3)	< 0.001
Median (IQR)		61.5 (46.0, 80.7)		57.7 (41.8, 77.6)	< 0.001
Offspring atopic outcomes					
Wheeze(∼ 7.5 years)	4696	499 (10.6%)	3331	361 (10.8%)	0.8
Asthma (∼ 7.5 years)	4648	576 (12.4%)	3290	393 (12.0%)	0.5
Eczema (∼ 7.5 years)	4686	772 (16.5%)	3328	535 (16.1%)	0.6
Hayfever (∼ 7.5 years)	4673	416 (8.9%)	3320	284 (8.6%)	0.6
Atopy (∼ 7 years)	3652	784 (21.5%)	2703	583 (21.6%)	0.9
Serum IgE (∼ 7 years) (ku/L)	2915	55.2 (18.5, 198)	2100	57.9 (20.3, 179)	0.39

* A test of the difference between those included in the analysis vs. others from the eligible cohort. *χ*^2^ test for categorical variables and *t*-test of log transformed variables for continuous variables.

† This *n* varies for each of our models because the *n* with each outcome is different and so here we report those with a maternal 25(OH) measure and at least one recorded outcome vs. those without any of the outcome measures.

‡ Adjusted for season.

Table 1 compares the main outcomes and exposures in the eligible sample included in the main analyses presented here and in those who were excluded because they did not have both an outcome measure and a measure of maternal 25(OH)D. Mothers of offspring with missing outcome data tended to have lower concentrations of 25(OH)D compared with those with available data, while there was no difference in the prevalence of any of the atopic outcomes between offspring of mothers with an exposure measure and those without. Mothers of offspring with missing data tended to be younger, of higher parity, from non-white ethnic groups, more likely to smoke and consume alcohol during pregnancy, from lower socio-economic groups, have higher levels of obesity, be less likely to be exposed to damp and mould in the home, have higher exposure to antibiotics and paracetamol during pregnancy and slightly lower reports of eczema, rhinoconjunctivitis, cold and flu and other infections. Eligible offspring with missing outcome data were less likely to be born at term and on average had a lower birth weight and a smaller head circumference (results not shown).

### Association between maternal 25(OH)D and covariables

Online Repository Table S2 shows the unadjusted associations of maternal characteristics with their 25(OH)D concentrations. Older and multiparous mothers had higher 25(OH)D concentrations. Mothers who were obese or underweight, exposed to tobacco smoke, and of non-white ethnicity had lower mean 25(OH)D concentrations. Home ownership, higher education levels and alcohol intake during pregnancy were associated with higher 25(OH)D concentrations. Slightly higher concentrations of 25(OH)D were seen among mothers that took antibiotics during pregnancy while there was no evidence of an association between maternal atopic history and 25(OH)D concentration. There was a modest correlation between season adjusted serum concentrations of 25(OH)D of pregnant mothers and those of their offspring (*r* = 0.16) Seasonally adjusted maternal 25(OH)D concentrations were strongly correlated with the predicted third trimester concentrations that were not adjusted for season (*r* = 0.88).

### Maternal 25(OH)D and asthma and atopic outcomes

**Fig. 2:**
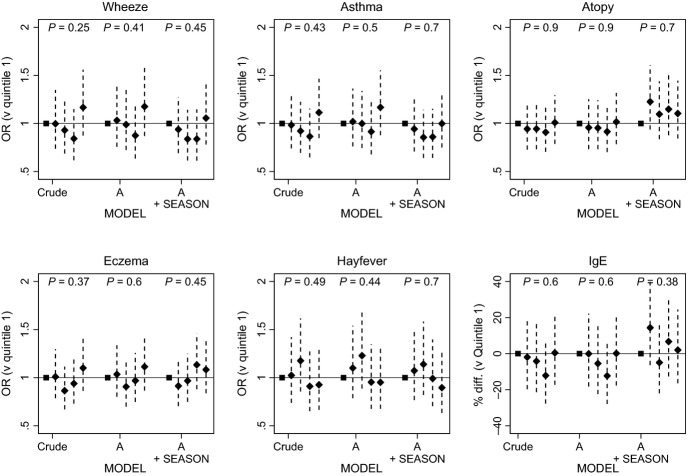
Minimally adjusted and adjusted odds ratios (OR) for offspring atopic outcomes comparing across quintiles of maternal 25(OH)D (min to 38 nmol/L (reference class); 38 to 52; 52 to 67; 67 to 89; 89 to max). For IgE the association is the mean% difference in IgE vs. the lowest (reference) quintile of maternal 25(OH)D. The *P*-values are a test of general association against the null of no effect. See Table S1 in the Online Repository for a description of the covariables included in each model.

Figure 2 shows the associations of maternal 25(OH)D with each of the asthma and atopic outcomes (these are also tabulated in Online Repository Table S3). Maternal 25(OH)D was not associated with any of the asthma/atopic outcomes. These null associations remained after adjustment for potential confounders and for season of measurement and there was no suggestion that offspring of mothers with very low (27.5 nmol/L) or high levels of circulating 25(OH)D were at an altered risk for atopic outcomes (see Figures S4 & S6 in Online Repository). In analyses using the longitudinal wheeze phenotypes, there was also little evidence to suggest any association with maternal 25(OH)D even when comparing persistent wheezers to infrequent or no wheezers (see Figure S9 in Online Repository).

### Maternal 25(OH)D and lung function

**Fig. 3:**
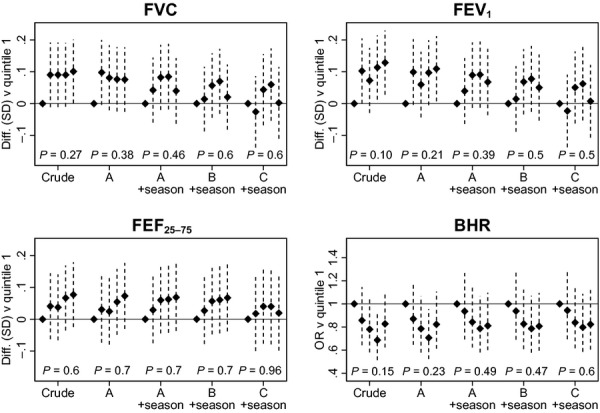
Minimally adjusted and adjusted mean difference in pulmonary function outcomes at 8 years (SD units) between quintiles of maternal 25(OH)D (min to 38 nmol/L (reference class); 38 to 52; 52 to 67; 67 to 89; 89 to max). The *P*-values are a test of general association against the null of no effect. See Table S1 in the Online Repository for a description of the covariables included in each model.

Figure 3 shows the associations of maternal 25(OH)D with each of the lung function outcomes (these are also tabulated in Online Repository Table S3). In unadjusted models, there was a weak suggestion of a threshold association between maternal 25(OH)D and FVC, FEV_1_ and BHR – offspring of mothers in the lowest quintile of 25(OH)D (< 38 nmol/L) tended to have poorer lung function and a higher odds of BHR compared with those from mothers in the higher quintiles. This pattern was attenuated after adjusting for potential maternal confounders and in particular for seasonality and the potential mediating effect of offspring growth. Furthermore, while this appeared to a be a qualitative dose–response relationship, there was actually no statistical evidence to support this overall relationship, and reanalysing 25(OH)D as categories of deficient, insufficient and sufficient 23 and using a threshold of 75 nmol/L also showed null results for a test of overall association (see Figures S5, S7 & S8 in online repository). As a post hoc analysis to further examine the suggestive trends in Fig. 3, we fitted equivalent models using age, height and sex standardized lung function outcomes at 15 years and there was no evidence that the patterns described above existed at this later age (see Figure S10 in Online Repository). There was a suggestion of an overall association between seasonally adjusted maternal 25(OH)D and FEV_1_ and FEF_25-75_ at 15 years although the pattern across quintiles was inconsistent, for example, compared with quintile 1, quintiles 2 and 4 had positive associations with FEV_1_ and FEF_25-75_, whereas quintiles 3 and 5 showed no association.

Our tests of an interaction between trimester of measurement and maternal 25(OH)D, did not provide evidence that associations differed depending upon which trimester of pregnancy 25(OH)D was assessed in (*P* > 0.4 for all interaction tests).

## Discussion

### Summary of main findings

In the largest study to date to examine the association of maternal 25(OH)D concentrations in pregnancy with offspring asthma, atopy and lung function, we found no evidence of a relationship between maternal 25(OH)D concentration and childhood wheeze, asthma, atopy, eczema, hayfever, IgE or pulmonary function, and no suggestion that either high or low concentrations of maternal 25(OH)D were associated with an increased risk of these outcomes. These findings were consistent regardless of adjustment for a range of potential confounding factors, including the seasonal influence on 25(OH)D.

### Strengths and limitations

The main strengths of this study are the large sample size, consideration of multiple atopic and respiratory outcomes, control for multiple potential confounders, incorporation of statistical methodology to account for missing data, use of several derivations of the maternal 25(OH)D exposure to check the robustness and sensitivity of our findings and to check the nature (e.g. linearity) of the association with each outcome.

Although we acknowledge the possibility of bias arising from loss to follow-up and missing data, we cannot conceive of a mechanism by which the association of maternal 25(OH)D and atopy or lung function would differ in the incomplete cases. Furthermore, our imputation model was relatively comprehensive in terms of the number of factors that predict the missing data and so would give unbiased results under an assumption that the data are missing at random. Serum 25(OH)D was assessed at only one time-point during pregnancy, this is unlikely to be representative of a mothers circulating levels over the whole of pregnancy and may have biased our results towards the null. Nonetheless, this single time-point assessment is the same as in other studies of this association and we would still have expected to find evidence of an association, if one existed, given the large sample size. Only a small proportion of mothers and offspring had deficient levels of 25(OH)D and the majority of mothers had levels below 75 nmol/L, so it is possible that we were unable to detect true associations operating at these levels. However, given the large sample size, and the range of vitamin D values, we would have expected to see some evidence of qualitative trends if this were the case and none was present. Furthermore, from a public health perspective maternal pregnancy vitamin D as a risk factor for future offspring atopy or respiratory health would only be of major importance if it was relevant to most of the population and not only those members at the extremes. Although we used carefully constructed models to derive each mother's exposure to 25(OH)D, these models were based on only one measure of 25(OH)D per mother taken at a random date during pregnancy. It is possible that this is a poor marker of vitamin D levels over pregnancy. However, we have examined this exposure in several ways and find no evidence that associations differ by trimester in which 25(OH)D was assessed and consistent null associations were found for all methods of 25(OH)D assessment. Serum 25(OH)D has been criticized as a proxy for vitamin D associations. For instance, it has been suggested that there is a need to distinguish between serum 25(OH)D and effects of vitamin D supplementation, the former being a prohormone representing a physiological state that is only relevant at extremely high or low levels, and the latter representing the non-physiological action of interest 4. Thus, the null findings here do not necessarily preclude important effects from supplementation, although trials have shown a positive and strong association between supplementation and 25(OH)D levels 26, 27.

### Comparison with other studies

The null findings seen in our study agree well with a recent analysis of maternal 25(OH)D measured in the 3rd trimester in a smaller UK cohort (Southampton Women's Study) which bears close resemblance to ALSPAC in terms of population and geographic location 13. Likewise, similar null associations have been reported from studies of maternal 25(OH)D and cord blood 25(OH)D in the United States 15 and Spain 12. Only one study reported a negative association with cumulative wheeze to 5 years, but that study also found no association with asthma 14. We could find no association with persistent wheeze to 6 years, even when examining those with and without atopy (results not shown). Although we could not directly investigate the association of maternal 25(OH)D with early life respiratory infections, the lack of association with transient early wheezing phenotype argues against a protective effect of prenatal vitamin D on respiratory viral infections in early childhood. It is possible that the only reported positive association of high maternal 25(OH)D with a higher risk of asthma in a UK cohort was a chance finding given the small sample size (< 500) 5.

The majority of positive findings come from studies of maternal dietary intake of vitamin D in pregnancy 8–10, 28. However, given that vitamin D intake is a poor proxy for total vitamin D status 11, apparent associations with dietary vitamin D may have been confounded by other dietary or non-dietary factors which had not been controlled for in those birth cohort studies. By way of analogy, in a study of vitamin D and adult lung function we found that, whilst there were apparent associations with dietary vitamin D intake, there was no evidence for an association with blood 25(OH)D concentrations 29. Alternatively previous positive dietary findings may have arisen through chance or represent publication bias. In support of this, it is notable that analysis of the association of maternal dietary vitamin D with offspring asthma, wheeze and atopy in both the Southampton Women's Survey 13 and ALSPAC 30 failed to demonstrate an association.

Although animal studies have shown that vitamin D is involved in normal fetal lung development 31, our study is in general agreement with other epidemiological studies that have considered later offspring lung function in the general population – two studies have reported null associations of maternal 25(OH)D 13, 32 or dietary vitamin D intake 32 with offspring lung function.

### Conclusion and implications

Our results, from the largest study to date, suggest the absence of a clinically important association between maternal 25(OH)D and offspring respiratory health or atopy. In particular, we have not found evidence to suggest that higher prenatal vitamin D status in pregnancy protects against the development of early childhood wheezing or later asthma. Whilst definitive evidence will only come from randomized trials of vitamin D supplementation during pregnancy, which are currently underway (e.g. see ISRCTN68645785) our results suggest that these interventions are unlikely to be beneficial for these outcomes. Reassuringly, our data also suggest that higher maternal vitamin D status in pregnancy is unlikely to increase the risk of atopy in the offspring.

## Supporting Information

**Figure S1.** Plot of trigonometric function to adjust maternal vitamin D for season.

**Figure S2.** Illustrative plot showing the derivation of the maternal 25(OH)D exposures.

**Figure S3.** Distribution of 25(OH)D in the mother.

**Figure S4.** Crude and adjusted odds ratios (OR) for offspring atopic outcomes comparing across thresholds of maternal 25(OH)D (< 50 nmol/L; 50–74.99 nmol/L (reference class); ≥ 75 nmol/L).

**Figure S5.** Crude and adjusted mean difference in pulmonary function outcomes (SD units) between thresholds of maternal 25(OH)D (< 50 nmol/L; 50–74.99 nmol/L (reference class); ≥ 75 nmol/L)

**Figure S6.** Crude and adjusted odds ratios (OR) for offspring atopic outcomes comparing across thresholds of maternal 25(OH)D (< 75 nmol/L (reference class); ≥ 75 nmol/L).

**Figure S7.** Crude and adjusted mean difference in pulmonary function outcomes (SD units) across a threshold of maternal 25(OH)D (< 75 nmol/L (reference), ≥ 75 nmol/L).

**Figure S8.** Crude and adjusted mean difference in pulmonary function outcomes (SD units) between thresholds of maternal 25(OH)D (< 27.5 nmol/L, 27.5–49.9 nmol/L; 50+ (reference class); ≥ 75 nmol/L).

**Figure S9**. Crude and adjusted associations between maternal 25(OH)D (nmol/L) and longitudinal wheeze phenotypes.

**Figure S10.** Crude and adjusted associations between maternal 25(OH)D (nmolL) and lung function at 15 y.

**Figure S11.** Complete case analysis.

**Figure S12.** Complete case analysis.

**Table S1.** Description of potential confounders and mediators and models

**Table S2.** Unadjusted associations of maternal characteristics with 25(OH)D in pregnancy. *N* = 5513^a^

**Table S3.** Coefficients for the results presented in Figures 2 and 3 of the main text. The coefficients (β) are odds ratios compared to the reference quintile unless stated**.** See supplementary Table S1 and methods in main text for a description of models

**Table S4.** Estimated probability of wheeze at each age by each latent class of wheeze.
