# Supplementary material for: Maternal 25-hydroxyvitamin D and its association with childhood atopic outcomes and lung function
Source: Clin Exp Allergy. 2013 Sep 16;43(10):1180–8. doi: 10.1111/cea.12172 (PMC3814422; doi:10.1111/cea.12172)
Supplement: Supplementary file 1 [file cea0043-1180-SD1.docx]

**Online Repository Material**

**Maternal 25-hydroxyvitamin D and its association with childhood atopic outcomes and lung function**

Authors:

Andrew K Wills PhD^1,2^; Seif O Shaheen PhD^3^; Raquel Granell PhD^2^; A John Henderson MD^2^; William D Fraser PhD^4^; Debbie A Lawlor PhD^1,2^

^1^ MRC Centre for Causal Analyses in Translational Epidemiology, University of Bristol, UK

^2^ School of Social and Community Medicine, University of Bristol, UK

^3^Centre for Primary Care and Public Health, Blizard Institute, Barts and The London School of Medicine and Dentistry, Queen Mary University of London, UK

^4^ Norwich Medical School, University of East Anglia, UK

1. **Supplementary methodological details**
   1. *Measurement of 25(OH)D in mothers and offspring*

Serum 25(OH)D in both mothers and offspring were measured in the same laboratory and using identical methods and protocols. 25(OH)D_3_ and 25(OH)D_2_ were measured with high performance liquid chromatography tandem mass spectrometry using internal standard in a laboratory meeting the performance target set by the Vitamin D External Quality Assessment Scheme (DEQAS) Advisory Panel for 25(OH)D assays. Inter-assay coefficients of variation for the assay were <10% across a working range of 1-250 ng/mL for both 25(OH)D_3_ and 25(OH)D_2_. The lower limit of detection was 0.5 ng/ml and the vast majority of mothers (74%), and 33% of offspring, had undetectable 25(OH)D_2_. Because so few pregnant mothers had detectable 25(OH)D_2_,_­_ and because previous studies have reported the associations of total 25(OH)D with offspring atopy, we calculated total 25(OH)D as the sum of 25(OH)D_3_ and 25(OH)D_2_ (allocating a value of 0.25ng/ml for 25(OH)D_2_ for those with levels below the detection limit) and used this in all analyses.

*1.2 Derivation of seasonal and gestational age adjusted 25(OH)D exposures:*

The following function was used to describe mean maternal total serum 25(OH)D over the period of data collection.

$$f\left( t \right)=\alpha+\sum_{h=1}^{4} \beta_{h}\sin\left( 2h\pi t \right)+\sum_{h=1}^{4} \theta_{h}\cos\left( 2h\pi t \right)$$

Where $\alpha{, \beta}_{h}\& \theta_{h}$ are estimated regression parameters and t is the date of blood sample (scaled to unit length covering the period of vitamin D assessment but ensuring seasonal periodicity at the ends). The number of terms $\beta_{h}\& \theta_{h}$was selected based on Wald tests and plots to check for over fitting at the ends of data. Figure S1 shows the predicted mean and the raw data.

This model and its residuals were used to (i) adjust total serum 25(OH)D to the midpoint of trimester 3 (34 weeks) for each individual, and (ii) to remove the seasonal pattern of 25(OH)D (the residuals from the model represents each mother’s level of 25(OH)D uncorrelated to their season of measurement). We adjusted to the midpoint of trimester 3 because the majority of mothers had blood taken in this trimester (52%).

**Figure E1. Plot of trigonometric function to adjust maternal vitamin D for season.**

25 (OH) D (nmol/l) was modelled on a natural logarithmic scale. Residuals were taken after back transformation of the predicted mean.


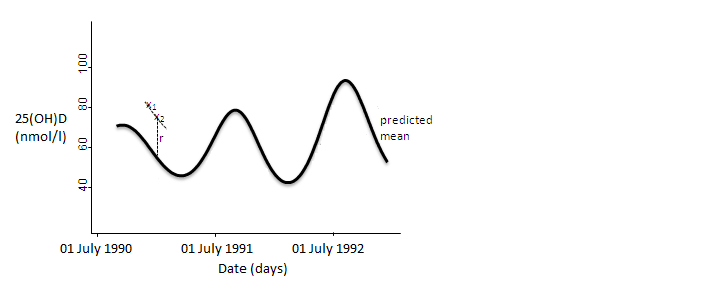


**Figure E2.** Illustrative plot showing the derivation of the maternal 25(OH)D exposures. A raw data point x_1_ is shown, x_2_ uses the predicted population mean and residual (r) to adjust x_1_ to the trimester midpoint x_2_. The season adjusted value the distance from the trigonometric (sinusoidal) regression line, as represented by r.

**Figure E3.** Distribution of 25(OH)D in the mother.

- 1. *Covariables*

Weight and height of mothers and children were measured in light clothing and without shoes. Weight was measured to the nearest 0.1kg using Tanita scales. Height was measured to the nearest 0.1cm using a Harpenden stadiometer. Birth weight was based on the routine clinical record, other neonatal anthropometric variables were measured within 1 day of birth by trained researchers. Gestational age was based on the last menstrual cycle provided it was not contradicted by results of an early ultrasound (<20weeks). All other variables were ascertained by questionnaires completed by the mothers.

*1.4 Additional details on the multiple imputation model*

All exposure, outcome and covariable variables that were included in any analysis model were included in the multiple imputation models. However, we did not impute any of the exposures or outcomes. Linear regression was use to impute for all continuous variables and multinomial and logit models were used for all categorical and binary variables, respectively. To ensure acceptable Monte Carlo error from imputation we created fifty imputed datasets. The figures in the appendix at the end of this file summarise the covariables, proportion of missing values and display the distribution of observed data and imputed data (from 10 of the imputed datasets).

Table E1. Description of potential confounders and mediators and models

| Model | | | Confounder | Values |
| --- | --- | --- | --- | --- |
| A | B | C | Male child | No, Yes |
| A | B | C | Mother’s age (y) | <20, 20-24, 25-29, 30-34, ≥35 |
| A | B | C | Prenatal tobacco exposure (maximum in pregnancy) | Not exposed, Passive only, Mother 1-9/d, Mother 10-19/d, Mother ≥20/d |
| A | B | C | Mother’s education level | <Ordinary level, Ordinary level, Advanced level/Higher degree |
| A | B | C | Housing tenure | Owned/mortgaged, Rented (public housing), Rented (non-public housing), Unknown/other |
| A | B | C | Parity | 0, 1, ≥2 |
| A | B | C | Maternal anxiety score | 0-4, 5-9, ≥10 |
| A | B | C | Mother’s ethnic origin | White, Nonwhite |
| A | B | C | Maternal BMI (kg/m2) | <18.5, 18.5-24.99, 25-29.99, ≥30 |
| A | B | C | Antibiotics at 20-32 wk gestation | Not reported, Reported |
| A | B | C | Alcohol units/wk over last 2 mo gestation | Never, <1/wk, ≥1/wk |
| A | B | C | Maternal paracetomol use at 20 weeks gestation | No, Yes |
| A | B | C | Maternal paracetomol use at 32 weeks gestation | No, Yes |
| A | B | C | Financial difficulties | None, Some, Many |
| A | B | C | Maternal asthma history | No, Yes |
| A | B | C | Maternal eczema history | No, Yes |
| A | B | C | Maternal rhinoconjunctivitis history | No, Yes |
| A | B | C | Maternal migraine history | No, Yes |
| A | B | C | Maternal cold/flu history | No, Yes |
| A | B | C | Maternal urinary infection history | No, Yes |
| A | B | C | Maternal other infections history | No, Yes |
| A | B | C | Younger siblings at 7 y | None, 1, ≥2 |
| A | B | C | Pets in first year | None, Cat or dog, Other pet |
| A | B | C | Breast-fed in first 6 mo | No, Yes |
| A | B | C | Child in day care at 8 mo | No, Yes |
| A | B | C | Damp, condensation, and mold | No, Yes |
| A | B | C | Child exposed to tobacco smoke at weekend | No, Yes |
|  | B | C | Gestational age (wk) | <37, 37-40, ≥41 |
|  | B | C | Birth weight (g) | <2500, 2500-2999, 3000-3499, 3500-3999, ≥4001 |
|  | B | C | Birth length (cm) | <48, 48-50.99, 51-53.99, ≥54 |
|  | B | C | Head circumference (cm) | <33, 33-34.99, 35-36.99, ≥37 |
|  | B | C | Child BMI at 7 y | <15.00, 15.00-17.49, 17.50-20.49, ≥20.5 |
|  |  | C | Child 25(OH)D (age and season adjusted) | Nmol/l |
|  |  |  |  |  |

1. **Additional results**

- 1. *Association between maternal 25(OH)D and potential confounders*

**Table E2. Unadjusted associations of maternal characteristics with 25(OH)D in pregnancy. N=5513** ^a^

|  |  | 25(OH)D (nmol/l): | Season adjusted 25(OH)D (nmol/l): |
| --- | --- | --- | --- |
| Potential confounder | level | Mean difference (95% CI) | Mean difference (95% CI) |
| Age | <20 | -8.3 (-14.2, -2.4) | -8.0 (-13.3, -2.6) |
|  | 20 to 24 | -5.3 (-7.8, -2.7) | -5.7 (-8.0, -3.3) |
|  | 25 to 30 | reference | reference |
|  | 30 to 34 | 2.2 (0.2, 4.2) | 1.9 (0.0, 3.7) |
|  | 35+ | 4.2 (1.4, 7.1) | 4.0 (1.4, 6.6) |
| BMI | <18.5 | -4.7 (-8.9, -0.5) | -6.8 (-10.6, -3.0) |
|  | 18.5 to 25 | reference | reference |
|  | 25 to 29.99 | -1.0 (-3.5, 1.5) | -1.7 (-4.0, 0.5) |
|  | 30+ | -5.6 (-9.7, -1.5) | -4.4 (-8.1, -0.7) |
| Parity | 0 | reference | reference |
|  | 1 | 3.8 (1.9, 5.7) | 4.2 (2.4, 5.9) |
|  | 2+ | 4.4 (2.0, 6.7) | 4.1 (2.0, 6.3) |
| Anxiety | 5 to 9 | -0.1 (-2.0, 1.8) | -0.1 (-1.8, 1.6) |
|  | 10+ | -1.4 (-4.4, 1.6) | -2.2 (-4.9, 0.5) |
| Ethnicity | White | reference | reference |
|  | Non-white | -18.5 (-25.1, -11.9) | -19.4 (-25.4, -13.5) |
| Education | < Ordinary | reference | reference |
|  | Ordinary | 3.3 (1.0, 5.5) | 3.0 (1.0, 5.1) |
|  | Advanced/Higher | 4.1 (1.9, 6.3) | 3.9 (1.9, 5.8) |
| Housing tenure | Owned/ mortgage | reference | reference |
|  | Rented (public) | -4.9 (-7.7, -2.0) | -5.8 (-8.4, -3.2) |
|  | Rented (non-public) | -3.5 (-7.1, 0.1) | -4.6 (-7.9, -1.4) |
|  | Other | -2.0 (-5.8, 1.9) | -3.7 (-7.2, -0.3) |
| Tobacco exposure | None | reference | reference |
|  | Passive only | -1.4 (-3.4, 0.5) | -1.4 (-3.1, 0.4) |
|  | Mother 1-9/d | -6.2 (-9.3, -3.0) | -5.4 (-8.3, -2.6) |
|  | Mother 10-19/d | -9.2 (-12.3, -6.1) | -9.4 (-12.2, -6.7) |
|  | Mother 20+ | -9.0 (-13.4, -4.7) | -9.6 (-13.6, -5.7) |
| Alcohol | None | reference | reference |
|  | 1/wk | 3.8 (1.9, 5.7) | 3.8 (2.0, 5.5) |
|  | >1/wk | 6.2 (3.8, 8.7) | 6.1 (3.9, 8.3) |

**Table E2 (cont). Association of maternal characteristics with 25(OH)D in pregnancy. N=5513**

|  |  | 25(OH)D (nmol/l): | Season adjusted 25(OH)D (nmol/l): |
| --- | --- | --- | --- |
| Potential confounder | level | Mean difference (95% CI) | Mean difference (95% CI) |
| Antibiotic use | No | reference | reference |
|  | Yes | 1.3 (-0.0, 2.7) | 1.3 (0.1, 2.5) |
| Asthma | No | reference | reference |
|  | Yes | 3.4 (-0.2, 7.1) | 2.4 (-0.9, 5.7) |
| Eczema | No | reference | reference |
|  | Yes | 0.6 (-2.1, 3.2) | -0.2 (-2.6, 2.2) |
| Rhinitis | No | reference | reference |
|  | Yes | 0.9 (-1.4, 3.1) | -0.3 (-2.3, 1.7) |

^a^ All analyses have had data missing on maternal characteristics imputed using multiple imputation.

*2.2 Analyses using recommended thresholds for maternal 25(OH)D:*

**Figure E4.** Crude and adjusted odds ratios (OR) for offspring atopic outcomes comparing across thresholds of maternal 25(OH)D (<50nmol/l; 50 to 74.99nmol/l (reference class); ≥75nmol/l). For IgE the association is the mean % difference in IgE across thresholds of maternal 25(OH)D. The p-values are a test of general association against the null of no effect. The covariables included in each model are described in supplementary Table S1.

**Figure E5.** Crude and adjusted mean difference in pulmonary function outcomes (SD units) between thresholds of maternal 25(OH)D (<50nmol/l; 50 to 74.99nmol/l (reference class); ≥75nmol/l). The p-values are a test of general association against the null of no effect. The covariables included in each model are described in supplementary Table E1.

**Figure E6.** Crude and adjusted odds ratios (OR) for offspring atopic outcomes comparing across thresholds of maternal 25(OH)D (<75nmol/l (reference class); ≥75nmol/l). For IgE the association is the mean % difference comparing. The covariables included in each model are described in supplementary Table E1.

**Figure E7.** Crude and adjusted mean difference in pulmonary function outcomes (SD units) across a threshold of maternal 25(OH)D (<75nmol/l (reference ), ≥75nmol/l). The p-values are a test of general association against the null of no effect. The covariables included in each model are described in supplementary Table E1.

**Figure E8.** Crude and adjusted mean difference in pulmonary function outcomes (SD units) between thresholds of maternal 25(OH)D (<27.5nmol/l, 27.5 to 49.9 nmol/l; 50+ (reference class); ≥75nmol/l). The p-values are a test of general association against the null of no effect. The covariables included in each model are described in supplementary Table E1.

**Table E3. Coefficients for the results presented in figures 2 and 3 of the main text. The coefficients (β) are odds ratios compared to the reference quintile unless stated.** See supplementary Table E1 and methods in main text for a description of models

|  |  | Crude |  |  | Model A |  |  | Model A + season |  |  |
| --- | --- | --- | --- | --- | --- | --- | --- | --- | --- | --- |
| outcome | s%R data? John will have a view.quintile | β (95%CI) | p | p(overall) | β (95%CI) | p | p(overall) | β (95%CI) | p | p(overall) |
| Wheeze | 1^st^ (ref) | 1 |  |  |  |  |  |  |  |  |
|  | 2^nd^ | 1.00 (0.74, 1.35) | 0.985 |  | 1.03 (0.76, 1.41) | 0.842 |  | 0.94 (0.69, 1.27) | 0.684 |  |
|  | 3^rd^ | 0.93 (0.69, 1.26) | 0.639 | 0.253 | 0.99 (0.72, 1.35) | 0.943 | 0.41 | 0.84 (0.62, 1.14) | 0.264 | 0.447 |
|  | 4^th^ | 0.84 (0.62, 1.15) | 0.28 |  | 0.88 (0.64, 1.21) | 0.414 |  | 0.84 (0.61, 1.15) | 0.271 |  |
|  | 5^th^ | 1.17 (0.87, 1.56) | 0.296 |  | 1.18 (0.87, 1.59) | 0.292 |  | 1.06 (0.78, 1.43) | 0.722 |  |
| Asthma | 1^st^ (ref) | 1 |  |  |  |  |  |  |  |  |
|  | 2^nd^ | 0.98 (0.74, 1.31) | 0.91 |  | 1.02 (0.76, 1.36) | 0.9 |  | 0.94 (0.71, 1.26) | 0.688 |  |
|  | 3^rd^ | 0.92 (0.69, 1.23) | 0.576 | 0.434 | 1.00 (0.75, 1.34) | 0.994 | 0.535 | 0.86 (0.64, 1.14) | 0.291 | 0.696 |
|  | 4^th^ | 0.87 (0.65, 1.15) | 0.325 |  | 0.92 (0.68, 1.23) | 0.56 |  | 0.86 (0.64, 1.15) | 0.314 |  |
|  | 5^th^ | 1.11 (0.85, 1.46) | 0.439 |  | 1.17 (0.88, 1.55) | 0.286 |  | 1.00 (0.75, 1.33) | 0.992 |  |
| Atopy | 1^st^ (ref) | 1 |  |  |  |  |  |  |  |  |
|  | 2^nd^ | 0.94 (0.73, 1.22) | 0.657 |  | 0.96 (0.73, 1.25) | 0.752 |  | 1.23 (0.94, 1.61) | 0.137 |  |
|  | 3^rd^ | 0.94 (0.73, 1.22) | 0.658 | 0.912 | 0.95 (0.73, 1.24) | 0.725 | 0.934 | 1.10 (0.84, 1.44) | 0.503 | 0.67 |
|  | 4^th^ | 0.91 (0.70, 1.17) | 0.461 |  | 0.92 (0.70, 1.19) | 0.511 |  | 1.15 (0.88, 1.50) | 0.314 |  |
|  | 5^th^ | 1.01 (0.79, 1.29) | 0.94 |  | 1.02 (0.78, 1.32) | 0.9 |  | 1.10 (0.84, 1.44) | 0.472 |  |
| Eczema | 1^st^ (ref) | 1 |  |  |  |  |  |  |  |  |
|  | 2^nd^ | 1.01 (0.79, 1.30) | 0.94 |  | 1.04 (0.80, 1.34) | 0.79 |  | 0.91 (0.70, 1.19) | 0.502 |  |
|  | 3^rd^ | 0.86 (0.67, 1.12) | 0.263 | 0.368 | 0.91 (0.70, 1.18) | 0.461 | 0.555 | 0.97 (0.75, 1.25) | 0.803 | 0.45 |
|  | 4^th^ | 0.94 (0.73, 1.21) | 0.629 |  | 0.97 (0.75, 1.26) | 0.815 |  | 1.13 (0.88, 1.46) | 0.334 |  |
|  | 5^th^ | 1.10 (0.86, 1.40) | 0.44 |  | 1.11 (0.87, 1.43) | 0.402 |  | 1.08 (0.84, 1.40) | 0.541 |  |

**Table E3 continued:**

|  |  | Crude |  |  | Model A |  |  | Model B |  |  |
| --- | --- | --- | --- | --- | --- | --- | --- | --- | --- | --- |
| outcome | quintile | β (95%CI) | p | p(overall) | β (95%CI) | p | p(overall) | β (95%CI) | p(overall) | p(overall) |
| Hayfever | 1^st^ (ref) | 1 |  |  |  |  |  |  |  |  |
|  | 2^nd^ | 1.02 (0.74, 1.42) | 0.887 |  | 1.10 (0.79, 1.54) | 0.577 |  | 1.07 (0.77, 1.50) | 0.681 |  |
|  | 3^rd^ | 1.18 (0.86, 1.62) | 0.317 | 0.492 | 1.23 (0.89, 1.70) | 0.216 | 0.443 | 1.14 (0.82, 1.58) | 0.435 | 0.664 |
|  | 4^th^ | 0.91 (0.65, 1.27) | 0.584 |  | 0.95 (0.68, 1.35) | 0.787 |  | 0.99 (0.70, 1.40) | 0.962 |  |
|  | 5^th^ | 0.93 (0.67, 1.29) | 0.649 |  | 0.95 (0.68, 1.34) | 0.776 |  | 0.90 (0.63, 1.27) | 0.538 |  |
| IgE* | 1^st^ (ref) | 0 |  |  |  |  |  |  |  |  |
|  | 2^nd^ | -1.93 (-19.76, 19.86) | 0.849 |  | -0.05 (-18.23, 22.18) | 0.996 |  | 14.38 (-6.38, 39.73) | 0.189 |  |
|  | 3^rd^ | -4.19 (-21.36, 16.72) | 0.671 | 0.626 | -5.47 (-22.41, 15.17) | 0.577 | 0.571 | -4.94 (-22.00, 15.85) | 0.616 | 0.389 |
|  | 4^th^ | -12.12 (-27.69, 6.80) | 0.194 |  | -12.31 (-27.93, 6.68) | 0.189 |  | 6.69 (-12.63, 30.27) | 0.525 |  |
|  | 5^th^ | 0.47 (-17.33, 22.11) | 0.962 |  | 0.16 (-17.65, 21.82) | 0.987 |  | 2.00 (-16.41, 24.48) | 0.845 |  |
| FVC† | 1^st^ (ref) | 0 |  |  |  |  |  |  |  |  |
|  | 2^nd^ | 0.09 (-0.01, 0.19) | 0.082 |  | 0.10 (-0.00, 0.20) | 0.059 |  | 0.04 (-0.06, 0.15) | 0.416 |  |
|  | 3^rd^ | 0.09 (-0.01, 0.19) | 0.081 | 0.273 | 0.08 (-0.02, 0.18) | 0.12 | 0.382 | 0.08 (-0.02, 0.18) | 0.115 | 0.463 |
|  | 4^th^ | 0.09 (-0.01, 0.19) | 0.077 |  | 0.08 (-0.02, 0.18) | 0.138 |  | 0.08 (-0.02, 0.19) | 0.108 |  |
|  | 5^th^ | 0.10 (0.00, 0.20) | 0.048 |  | 0.08 (-0.03, 0.18) | 0.144 |  | 0.04 (-0.06, 0.14) | 0.45 |  |
| FEV_1_† | 1^st^ (ref) | 0 |  |  |  |  |  |  |  |  |
|  | 2^nd^ | 0.10 (0.00, 0.21) | 0.05 |  | 0.10 (-0.00, 0.20) | 0.059 |  | 0.04 (-0.06, 0.14) | 0.456 |  |
|  | 3^rd^ | 0.07 (-0.03, 0.18) | 0.164 | 0.105 | 0.06 (-0.04, 0.16) | 0.257 | 0.212 | 0.09 (-0.01, 0.19) | 0.089 | 0.386 |
|  | 4^th^ | 0.11 (0.01, 0.21) | 0.028 |  | 0.10 (-0.00, 0.20) | 0.061 |  | 0.09 (-0.01, 0.20) | 0.086 |  |
|  | 5^th^ | 0.13 (0.03, 0.23) | 0.012 |  | 0.11 (0.01, 0.21) | 0.036 |  | 0.07 (-0.04, 0.17) | 0.201 |  |

**Table E3 continued:**

|  |  | Crude |  |  | Model A |  |  | Model B |  |  |
| --- | --- | --- | --- | --- | --- | --- | --- | --- | --- | --- |
| outcome | quintile | β (95%CI) | p | p(overall) | β (95%CI) | p | p(overall) | β (95%CI) | p(overall) | p(overall) |
| FEF_25-75_† | 1^st^ (ref) | 0 |  |  |  |  |  |  |  |  |
|  | 2^nd^ | 0.04 (-0.06, 0.14) | 0.442 |  | 0.03 (-0.07, 0.14) | 0.568 |  | 0.03 (-0.08, 0.13) | 0.588 |  |
|  | 3^rd^ | 0.04 (-0.07, 0.14) | 0.479 | 0.617 | 0.02 (-0.08, 0.13) | 0.645 | 0.676 | 0.06 (-0.04, 0.16) | 0.264 | 0.679 |
|  | 4^th^ | 0.07 (-0.04, 0.17) | 0.2 |  | 0.05 (-0.05, 0.16) | 0.301 |  | 0.06 (-0.04, 0.17) | 0.244 |  |
|  | 5^th^ | 0.08 (-0.02, 0.18) | 0.138 |  | 0.07 (-0.03, 0.18) | 0.164 |  | 0.07 (-0.04, 0.17) | 0.198 |  |
| BHR | 1^st^ (ref) | 1 |  |  |  |  |  |  |  |  |
|  | 2^nd^ | 0.86 (0.64, 1.15) | 0.298 |  | 0.87 (0.65, 1.17) | 0.364 |  | 0.94 (0.69, 1.27) | 0.67 |  |
|  | 3^rd^ | 0.78 (0.58, 1.05) | 0.1 | 0.147 | 0.79 (0.58, 1.07) | 0.12 | 0.234 | 0.84 (0.62, 1.14) | 0.267 | 0.494 |
|  | 4^th^ | 0.69 (0.51, 0.92) | 0.011 |  | 0.71 (0.52, 0.96) | 0.024 |  | 0.79 (0.58, 1.07) | 0.122 |  |
|  | 5^th^ | 0.83 (0.62, 1.10) | 0.191 |  | 0.82 (0.61, 1.11) | 0.197 |  | 0.81 (0.60, 1.10) | 0.175 |  |

*Mean difference in % versus the reference exposure quintile (lowest)

† Mean difference in standard deviation scores versus the reference exposure quintile (lowest)

*2.3 Longitudinal latent class wheeze phenotypes*

At 6months (m), 1y 6m, 2y 6m, 3y 6m, 4y 9m, 5y 9m and 6y 7m, each mother was asked the following 2 questions; Did the child wheeze during the last 12 months (6 months at baseline)?Did the child wheeze and whistle during the last 12 months? A new variable was created for each age to indicate wheeze if their mother had responded yes to any of these questions. These new variables were entered into a latent class analysis (1) to establish whether there was any substructure to these responses and hence whether certain phenotypes could be defined. All individuals with at least 2 responses were included with missing data treated under a missing at random assumption. A model with 6 classes was deemed the best fit based on various fit indices and model entropy. Table S4 shows the composition of each class by the probability of wheeze at each age.

Associations between maternal 25(OH)D and each longitudinal wheeze class were then estimated using multinomial logistic regression. The uncertainty in class assignment was taken into account by weighting each individual’s class membership by their predicted probability of class membership. The Never/infrequent class was used as the reference category.

Table E4 Estimated probability of wheeze at each age by each latent class of wheeze.

|  | Class |  |  |  |  |  |
| --- | --- | --- | --- | --- | --- | --- |
| Age | Never or Infrequent (61.2% | Transient early (12.8%) | Prolonged early (9.8%) | Intermediate onset (2.4%) | Late onset (6.3%) | Persistent 7.5%) |
| 0y 6m | 0.11 | 0.63 | 0.49 | 0.1 | 0.22 | 0.65 |
| 1y 6m | 0.07 | 0.71 | 0.63 | 0 | 0.24 | 0.93 |
| 2y 6m | 0.05 | 0.33 | 0.35 | 0.55 | 0.2 | 0.89 |
| 3y 6m | 0.03 | 0.03 | 0.58 | 0.97 | 0.19 | 0.89 |
| 4y 9m | 0.03 | 0.07 | 0.41 | 1 | 0.51 | 0.92 |
| 5y 9m | 0.02 | 0.04 | 0.05 | 0.9 | 0.78 | 0.88 |
| 6y 7m | 0.02 | 0.06 | 0.05 | 0.84 | 0.55 | 0.8 |

**Figure E9**. Crude and adjusted associations between maternal 25(OH)D (nmol/l) and longitudinal wheeze phenotypes. Odds ratios are for respective class membership versus the reference class of infrequent wheeze and given in quintiles of 25(OH)D (min to 38nmol/l (reference group); 38 to 52; 52 to 67; 67 to 89; 89 to max). The p-values are for a test of general association across quintiles versus the reference class. The covariables included in each model are described in supplementary Table E1.

*2.4 Post hoc analysis: association with pulmonary function at 15 years*

To test whether the associations between maternal 25(OH)D and pulmonary function at 8 years were still evident at later ages, we repeated analysis using pulmonary measurements made at the 15 year clinic (mean age 15.5 (SD 0.29). Measurements were made using a Vitalograph 2120 spirometer (Maids Moreton, UK) according to ATS/ERS criteria. The equipment was calibrated at the start of each day with a 3L calibration syringe to ensure volume accuracy to within +/-3.5% and linearity of the pneumotachometer was checked weekly at different flow rates using the same accuracy thresholds. Measurements were made with the child seated and wearing a nose clip. The best measurements from three reproducible flow-volume curves were used for analyses. Flow-volume curves were reviewed by one respiratory physician to ensure adherence to standards. Forced expiratory volume in 1s (FEV_1_), forced vital capacity (FVC) and mid forced expiratory flow (FEF_25–75_) were converted to gender, age and height adjusted standard deviation units.

**Figure E10.** Crude and adjusted associations between maternal 25(OH)D (nmolL) and lung function at 15y. The mean difference and 95% CI in lung function (SD units) are plotted for each quintile of maternal 25(OH)D with respect to the 1^st^ quintile (min to 38nmol/l (reference group); 38 to 52; 52 to 67; 67 to 89; 89 to max). The covariables included in each model are described in supplementary Table E1.

*2.5 Results from complete case analysis*

**Figure E11. Complete case analysis:**

Crude and adjusted odds ratios (OR) for offspring atopic outcomes comparing across quintiles of maternal 25(OH)D (min to 38nmol/l (reference class); 38 to 52; 52 to 67; 67 to 89; 89 to max). For IgE the association is the mean % difference in IgE across quintiles of maternal 25(OH)D. The p-values are a test of general association against the null of no effect. The covariables included in each model are described in supplementary Table E1. All models used the same sample, n’s are given within each plot.

**Figure E12. Complete case analysis:**

Crude and adjusted mean difference in pulmonary function outcomes at 8.5 years between quintiles of maternal 25(OH)D (min to 38nmol/l (reference class); 38 to 52; 52 to 67; 67 to 89; 89 to max). The p-values are a test of general association against the null of no effect. The covariables included in each model are described in supplementary Table E1. All models used the same sample, n’s are given within each plot.

Reference List

(1) Henderson J, Granell R, Heron J, Sherriff A, Simpson A, Woodcock A et al. Associations of wheezing phenotypes in the first 6 years of life with atopy, lung function and airway responsiveness in mid-childhood. Thorax 2008 November 1;63(11):974-80.

**Appendix. Distributions of observed (obs) and imputed missing data for each of the covariables:**

For clarity, the distributions are shown only for the first 10 imputed datasets. Where no data were missing only the observed data are shown. The n and % of missing values for each variable are reported in the bottom corner of the plot.
